# Values, challenges, and responses associated with high-priced potential cures: perspectives of diverse stakeholders in South Korea

**DOI:** 10.1186/s12962-024-00527-2

**Published:** 2024-03-04

**Authors:** Jihyung Hong, Eun-Young Bae, Hye-Jae Lee, Tae-Jin Lee, Philip Clarke

**Affiliations:** 1 Department of Healthcare Management, Gachon University, Seongnam, South Korea; 2 College of Pharmacy, Gyeongsang National University, 501 Jinju-daero, 52828 Jinju, South Korea; 3 Department of Environmental Health, Korea National Open University, Seoul, South Korea; 4 Department of Public Health Science, Graduate School of Public Health, Seoul National University, Seoul, South Korea; 5 Health Economics Research Centre, Nuffield Department of Population Health, University of Oxford, Oxford, UK

**Keywords:** High-priced, Potential cures, Gene therapies, Managed entry agreements

## Abstract

**Background:**

The emergence of high-priced potential cures has sparked significant health policy discussions in South Korea, where the healthcare system is funded through a single-payer National Health Insurance model. We conducted focus group interviews (FGIs) and accompanying surveys with diverse stakeholders to comprehensively understand related issues and find better solutions to the challenges brought by these technologies.

**Methods:**

From October to November 2022, 11 FGIs were conducted with stakeholders from various sectors, including government payers, policy and clinical experts, civic and patient organisations, and the pharmaceutical industry, involving a total of 25 participants. These qualitative discussions were supplemented by online surveys to effectively capture and synthesise stakeholder perspectives.

**Results:**

Affordability was identified as a critical concern by 84% of stakeholders, followed by clinical uncertainty (76%) and limited value for money (72%). Stakeholders expressed a preference for both financial-based controls and outcome-based pricing strategies to mitigate these challenges. Despite the support for outcome-based refunds, payers raised concerns about the feasibility of instalment payment models, whether linked to outcomes or not, due to the specific challenges of the Korean reimbursement system and the potential risk of ‘cumulative liabilities’ from ongoing payments for previously administered treatments. In addition, the FGIs highlighted the need for clear budgetary limits for drugs with high uncertainties, with mixed opinions on the creation of special silo funds (64.0% agreement). Less than half (48%) endorsed the use of external reference pricing, currently applied to such essential drugs in South Korea. A significant majority (84%), predominantly non-pharma stakeholders, advocated for addressing cost-effectiveness uncertainty through re-assessment once long-term clinical data become available.

**Conclusions:**

This study uncovers a broad agreement among stakeholders on the need for more effective value assessment methodologies for high-priced potential cures, stressing the importance of more robust and comprehensive re-assessment supported by long-term data collection, rather than primarily relying on external reference pricing. Each type of stakeholders exhibited a cautious approach to their specific uncertainties, suggesting that new funding strategies should accommodate these uncertainties with predefined guidelines and agreements prior to the initiation of managed entry agreements.

**Supplementary Information:**

The online version contains supplementary material available at 10.1186/s12962-024-00527-2.

## Background

Recent launches of potential cures, such as cell and gene therapies, have put these technologies at the forefront of the pharmaceutical policy agenda in South Korea, as in many other countries [1, 2]. While these technologies have the potential to provide substantial, long-lasting health benefits to patients, they also pose extreme challenges for healthcare payers. Value-based pricing of these technologies often leads to very high prices well beyond traditional price ranges, yet their values at launch are likely highly uncertain due to immature clinical data [3]. Expedited regulatory programmes, introduced to help accelerate the speed of marketing authorisation (MA) for potentially life-saving technologies [4], further limit clinical evidence available to payers for pricing and reimbursement (P&R) decision-making on these technologies.

Given the budgetary impact and uncertainty about long-term health outcomes, healthcare payers have increasingly adopted managed entry agreements (MEAs), albeit not specific to high-priced potential cures [1]. Financial-based agreements (FBA), such as rebates/discounts and price-volume arrangements, have already been widely employed in many countries because they are believed to be administratively simple and offer ‘tangible’ budget gains for the payers [1, 5]. These budget savings from confidential FBAs could be even larger for sizable payers and countries with greater purchasing power [6]. However, these schemes alone cannot adequately address uncertainties surrounding the value of these technologies, as they are ultimately budget-containing tools [7]. Performance-based agreements (PBA), such as outcome-linked payments and coverage with evidence development (CED), have also been explored to better deal with those uncertainty challenges. However, the use of PBAs, possibly conceptually more attractive than FBAs, has been limited as they are more costly and administratively burdensome, as suggested in the pinioning experience of the Italian Medicines Agency (AIFA) and other countries [7]. Despite significant investments in real-world data collection (e.g., establishing registries), the AIFA’s outcome-linked payment has been criticised because it has relied primarily on surrogate endpoints and made little contribution to budget savings [8–10]. Other countries, such as the Netherlands and Sweden, shared similar experiences with CED [1]. They reported difficulties executing CED arrangements, which made them gradually move towards other approaches [1]. Nevertheless, recent literature suggests that PBAs, particularly outcome-linked payments, have become very appealing, at least for high-priced potential cures [11–13].

Payers have also explored new pricing mechanisms to better deal with the nature of these technologies, which often have high upfront costs, especially for one-time or short-term treatments, yet their health benefits likely accrue over a longer period. The instalment type of payments, whether or not linked to outcomes, is one such example suggested to mitigate the initial budget impacts and correct the misalignment between payments and benefits [14]. However, the feasibility of any new pricing mechanism should be assessed and adjusted in the context of specific health systems. For instance, the continuity of instalment payments should be carefully considered in a health system like the US, where patients typically switch insurers over time [15]. Therefore, this type of payment in such health systems requires special payer incentives or regulations to ensure that future payers commit to future payments. Likewise, other considerations will certainly be required when introducing new types of MEAs and pricing mechanisms in different health systems.

South Korea, where healthcare is publicly funded through the National Health Insurance (NHI) (a single-payer system) but primarily provided by private healthcare providers, has begun to actively seek new P&R approaches for these potential cures, especially after the introduction of Kymriah (for B-cell acute lymphoblastic leukaemia and diffuse large B cell lymphoma) and Zolgensma (for spinal muscular atrophy) with record-high prices in 2021/2022 [16, 17]. Currently in South Korea, manufacturers are, in general, required to submit cost-effective analysis (CEA) data for listing their new drug on the NHI formulary at premium prices. However, new drugs under high unmet needs (i.e., targeting cancer or rare, life-threatening conditions without therapeutic alternatives) can be introduced through MEAs. While some anticancer or orphan drugs have already been introduced through MEAs (mostly FBAs) with or without CEA data, the recent launch of high-priced potential cures with limited clinical and economic evidence has re-ignited affordability and uncertainty issues in South Korea.

We conducted focus group interviews (FGIs) and accompanying surveys with a diverse group of key stakeholders to gain a more comprehensive understanding of related issues and help develop joint solutions to the challenges brought by high-priced potential cures.

## A brief description of pharmaceutical policy in South Korea

In 2006, South Korea implemented a positive listing system, mandating drug manufacturers to provide CEA data to justify premium pricing for their new drugs on the NHI formulary. The Health Insurance Review and Assessment Service (HIRA) evaluates these submissions, issuing reimbursement recommendations primarily based on clinical and cost-effectiveness. Notably, drugs with an Incremental Cost-Effectiveness Ratio (ICER) below KRW 25 million (approximately US$20,800 [18]), and KRW 50 million for anti-cancer drugs, were implicitly considered cost-effective. However, HIRA has recently clarified it does not adhere to explicit or implicit ICER thresholds but considers past ICER outcomes in its decisions [19].

To improve access to medications for conditions with significant unmet medical needs, such as rare and life-threatening diseases, the Korean government introduced risk-sharing agreements (RSAs), a form of MEAs, in 2013. RSAs encompass various models, including refunds, expenditure caps, utilisation caps per patient, free initial treatment, and outcome-based pricing, with refunds being most common [18]. Drugs under RSA are nonetheless required to submit CEA data for premium pricing.

In response to claims that stringent CEA data requirements were delaying access to essential medications, the government introduced a CEA waiver policy in 2015 for anticancer or orphan drugs aiming at a small patient population. Eligibility for this policy requires satisfying specific criteria [20]: limited treatment options, challenges in generating robust evidence (e.g., data from single-arm studies), and approval in at least three reference countries (A7 countries: France, Germany, Italy, Japan, Switzerland, the UK, and the USA, with Canada added in December 2022, expanding the group to A8). The pricing under this policy reflects the adjusted list prices in these reference countries, though such drugs remain subject to MEAs, often including an expenditure cap and additional requirements. More information on this policy can be found in the supplementary online materials (Appendix 1).

## Materials and methods

### Study design and participants

From October to November 2022, we conducted 11 virtual, semi-structured FGIs/individual interviews with a diverse group of key stakeholders to explore their perspectives on the values, challenges, and effective responses to high-priced potential cures. Each FGI lasted approximately 2 h, tailored to accommodate the stakeholders’ schedules. Following the FGIs, online surveys—developed concurrently with the FGI questionnaires—were also administered to quantitatively summarise their opinions in a consistent manner.

Twenty-five stakeholders, through purposive sampling, were included in these interviews and accompanying surveys drawn from diverse groups as follows: **(i) government officials (single payers)** in charge of drug P&R and health technology assessment (HTA) policies and processes, composed of those from the Ministry of Health and Welfare (*n* = 1), the National Health Insurance Service (NHIS [a single payer], *n* = 2) and the Health Insurance Review and Assessment Service (HIRA [HTA body], *n* = 2); **(ii) health economics and policy experts** (*n* = 6) with more than ten years of experience in P&R and HTA; **(iii) clinical experts** (*n* = 3) who have experience using high-priced potential cures in their practice; **(iv) civic group representatives** (*n* = 7) nominated by major organisations, including patient groups (*n* = 2), workers’ and employers’ organisations (*n* = 2), consumer groups (*n* = 2), and one from another category (*n* = 1); and **(v) pharma P&R staff** from both local (*n* = 2) and global (*n* = 2) pharma with MEA experiences for high-priced drugs.

Each of the 11 interviews (two expert groups, three civic groups, four government groups and two pharma groups) was conducted with one to five participants. The size of interview groups was purposely kept small to encourage the active participation of all stakeholders, especially given the virtual environment, and to enable expressions of stakeholders’ frank opinions without any conflicting interests across groups or organisational hierarchies. For instance, interviews were separately conducted for each type of government organisation.

Meeting materials summarising the characteristics and issues of high-priced potential cures, together with other countries’ responses, were provided in advance. A further verbal briefing was also provided at the beginning of each interview. We did not clearly define high-priced potential cures (e.g., price thresholds and drug categories) but provided the key characteristics of these drugs (see Appendix 2 for further details [online supplements]) with recent P&R cases (e.g., Kymriah and Zolgensma). The research team members led the interviews because topics required subject matter knowledge. Stakeholders were asked to freely discuss their experiences and opinions about the following topics: the values and issues of high-priced potential cures (opening questions) and possible solutions to the affordability and uncertainty challenges (key questions). We did not evenly divide the length of discussion for each topic– it was rather tailored to the type of stakeholders.

The interviews and surveys were approved by the Institutional Review Board of Seoul National University, and written informed consent was obtained from all participants.

### Data collection and analysis

All interviews were audio recorded and professionally transcribed verbatim. The key messages were shared among the research team following each interview and verified with full interview transcripts. Nonetheless, stakeholder views were summarised mainly based on the online survey data in this text, backed up with detailed reasons and experiences obtained from the FGIs. This approach was taken to minimise the involvement of the authors’ potential subjective judgements when presenting and concluding the study findings.

As in the FGIs, the surveys had four themes related to high-priced potential cures: *i*) values (additional societal values, components of cures, factors to consider in P&R decisions), *ii*) issues (affordability, clinical uncertainty, limited value for money, lack of transparency in pricing, and early patient access), *iii*) responses to the budget impact challenge (conventional financial controls, new pricing mechanisms, special silo funds, and other), and *iv*) responses to the uncertainty challenge (response mechanisms, type of uncertainty, and re-assessment-related issues). Many of these questions asked the level of agreement with a five-point Likert scale (1 = strongly disagree, 2 = disagree, 3 = neither agree nor disagree (fair), 4 = agree, 5 = strongly agree). Descriptive statistics were used to summarise the percentages of agreements (i.e., agree/strongly agree) with supplementary mean and standard deviation (SD). As pharma are likely to have their own interests, which are likely different from the views of other stakeholders, the level of agreement was also summarised by the type of stakeholders (pharma [*n* = 4] vs. non-pharma [*n* = 21]), using mean with SD.

## Results

### Values and concerns associated with high-priced potential cures

**Fig. 1:**
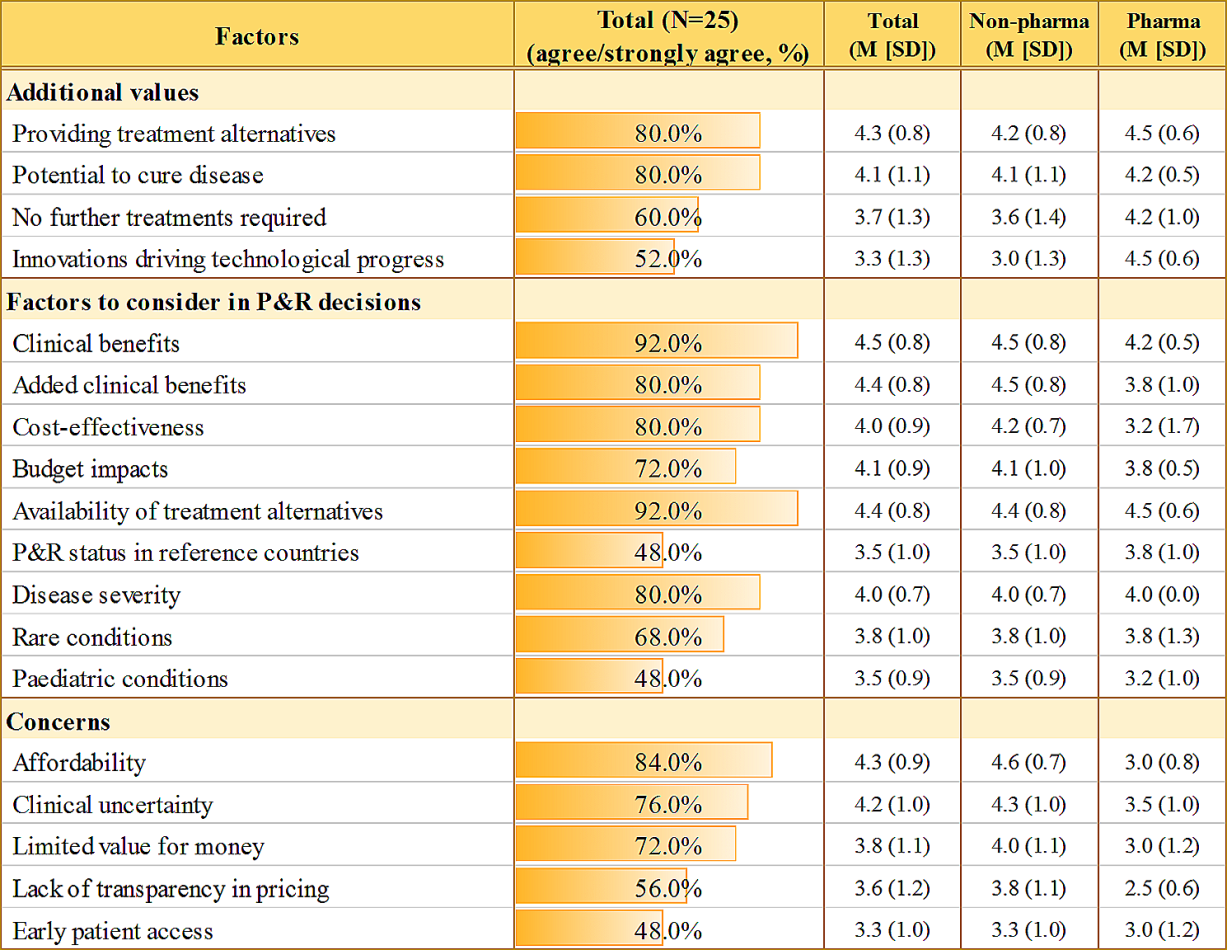
Values and concerns associated with high-priced potential cures. *Note* Level of agreement on the above factors was surveyed using a five-point likert scale (1 = strongly disagree, 5 = strongly agree), following focus group interviews to quantitatively summarise the stakeholders’ perspectives in a consistent manner. Values were presented as either percentages or mean (M) with standard deviation (SD), as indicated. Non-pharma includes policy & clinical experts, government payers, civic groups, and patient groups. *Abbreviations* P&R, pricing and reimbursement

**Fig. 2:**
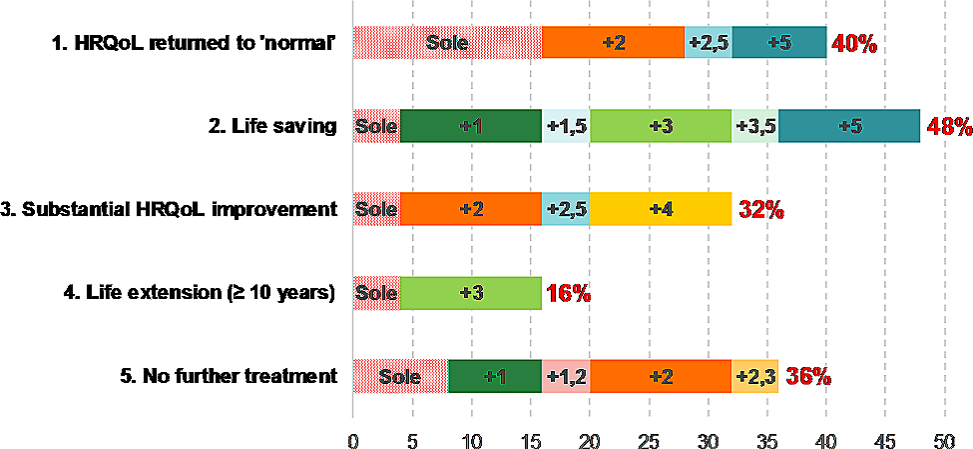
Concept components for cures. *Abbreviations* HRQoL, health-related quality of life. *Note* Each bar indicates % of respondents agreed to the corresponding component with (as indicated with a plus sign [e.g., “+2” implies “together with 2.life-saving”]) and without other components (sole). For instance, 40% of respondents considered returning HRQoL to ‘normal’ as essential, with variations: 16% as the sole criterion, 12% with life-saving (+ 2), 8% with no further treatment (+ 5), and 4% with life-saving and no further treatment (+ 2, 5).

Twenty-five key stakeholders from diverse groups participated in the semi-structured FGIs and completed the accompanying online surveys, with no missing data. The stakeholders were first asked to freely discuss the values and concerns associated with high-priced potential cures. Overall, they recognised the high value of these technologies, mainly because they can offer promising opportunities for patients who would otherwise have no treatment options (see Fig. 1). Notably, stakeholders tended to understand ‘cure’ as a significant step-change. Only one in 25 stakeholders answered that ‘cure’ requires returning to ‘normal’ (level of the same age) in terms of both health-related quality of life (HRQoL) and life years (LY) without further treatments (Fig. 2). The rest agreed on the less strict definitions for cures, such as substantial HRQoL improvement (32%) and life extension of at least ten years (16%) with/without other components.

We also asked what factors should be considered in the P&R decisions for high-priced potential cures (Fig. 1). Nearly everyone agreed to clinical benefits (90%) and availability of treatment alternatives (90%), followed by added clinical benefits (80%), cost-effectiveness (80%) and disease severity (80%). Relatively fewer stakeholders agreed to rarity (68%), paediatric conditions (48%) and the P&R status in reference countries (48%).

Despite these values recognised, stakeholders most cited affordability as a concern of high-priced potential cures (84%), followed by clinical uncertainty (76%) and limited value for money (72%). Relatively fewer agreed that a lack of transparency in pricing (56%) and patient access problems (48%) are a concern (Fig. 1).

### Ways to manage the budget impacts of high-priced potential cures

#### Conventional FBAs and additional outcome-based pricing arrangements

**Fig. 3:**
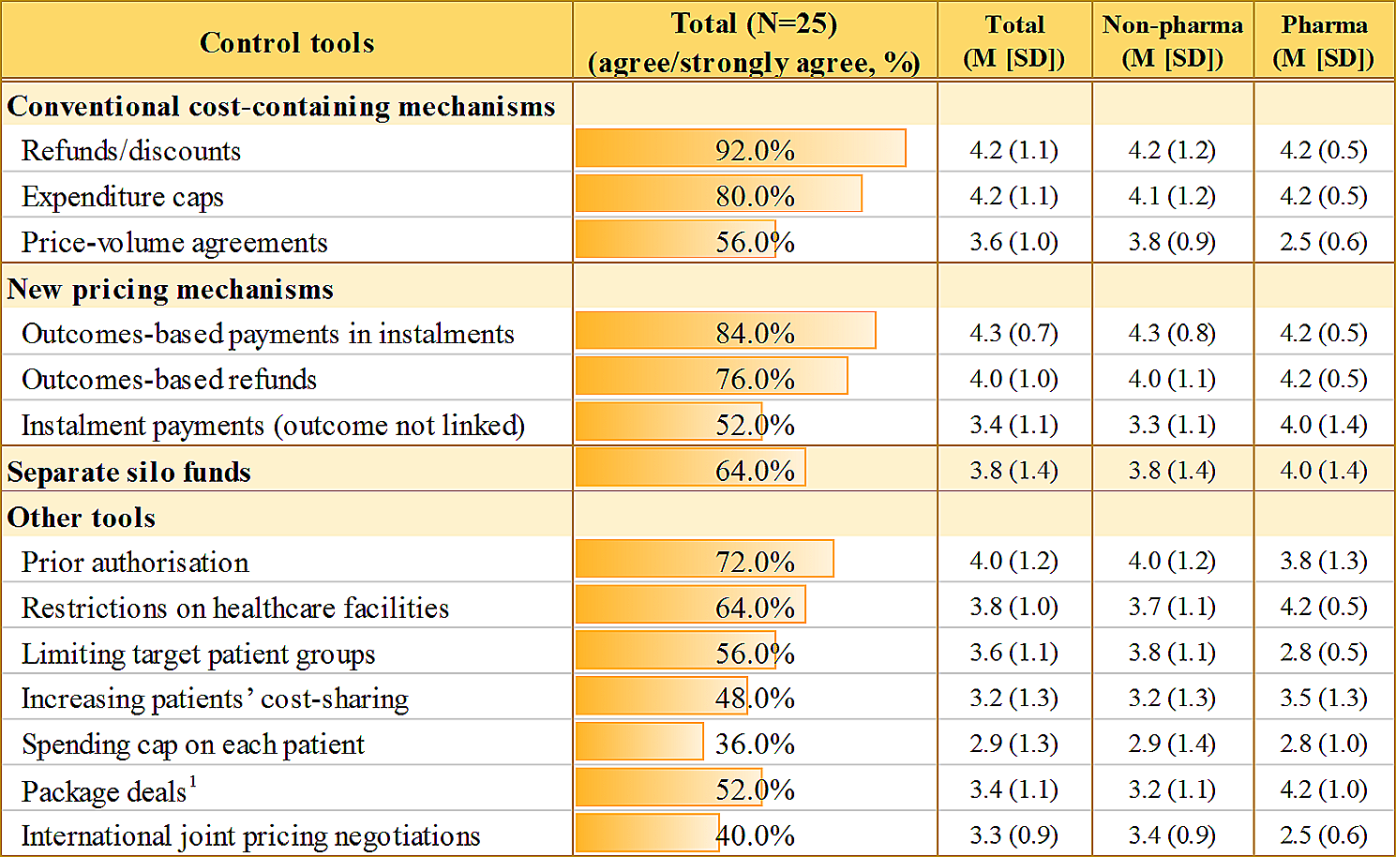
Responses to the affordability challenges of high-priced potential cures. *Note* Level of agreement on the above factors was surveyed using a five-point likert scale (1 = strongly disagree, 5 = strongly agree), following focus group interviews to quantitatively summarise the stakeholders’ perspectives in a consistent manner. Values were presented as either percentages or mean (M) with standard deviation (SD), as indicated. Non-pharma includes policy & clinical experts, government payers, civic groups, and patient groups. ^1^Package deals refer to arrangements that permit charging higher prices for potential cures that have limited clinical evidence at the time of launch, while simultaneously lowering the prices for other medications produced by the same manufacturers

A majority of stakeholders agreed to conventional FBAs such as rebates/discounts (92.0%) and expenditure caps (80.0%) as a default mechanism to control the budget impacts of high-priced potential cures, although far fewer (56.0%), especially pharma, agreed to the use of price-volume agreements (Fig. 3). Some experts even argued for the extensive application of FBAs allowing for dual pricing because net prices (real prices) protected under confidentiality agreements would not affect prices in neighbouring countries like China (external reference pricing), making it easier for manufacturers to agree to Korea’s net price cuts.

Many stakeholders, including pharma, also mentioned outcome-linked payments as a new payment model as long as outcomes can be clearly defined, measured, and interpreted without disputes. The percentage of agreement was even higher for outcomes-based payments in instalments (i.e., payments initiated if predetermined goals are met) (84%) than outcomes-based refunds (i.e., refunds issued if goals are missed) (76%) since the former, in principle, completely removes the uncertainty associated with implementing future refunds (e.g., due to manufacturers’ bankruptcy). It can also nicely mitigate the high upfront costs of one-time or short-term treatments. However, payers expressed practical concerns over outcome-based payments in instalments. They mentioned that any pricing models involving instalment payments, whether or not linked to outcomes, are likely possible only if healthcare providers are involved in such MEA contracts because they are the ones who purchase treatments and need to get reimbursed by the NHIS in South Korea. They expressed greater concerns over ‘cumulative liabilities’ from ongoing payments for previously administered treatments than over the high upfront costs of these treatments. This is because such deferred payments could limit flexibility in responding to future financial NHI conditions.

The NHI collects contributions and uses them for that year. Let’s assume that we make a payment in two or three instalments. What if the NHI financial condition is not good when these future instalments need to be made? We need to consider future NHI affordability. (a payer)

Meanwhile, the government commented positively on outcome-based refunds since any rebates and refunds can be directly taken from the manufacturers and treated as ‘additional revenues of the NHIS’. As for instalment payments not linked to outcomes, the level of agreement was relatively lower (52%), even among non-government stakeholders. Since the government is a single payer in South Korea and the target population is not large, fewer stakeholders appreciated the need for such pricing models only to spread out the high upfront costs of one-time or short-term treatments over several years.

#### Special silo funds

Special silo funds were one of the mechanisms most frequently mentioned during the FGIs, although not all agreed to the funds (20% disagreed, 64% agreed). Those who agreed tended to appreciate the need for setting clear budget limits for those drugs having significant budget impact and uncertainty implications.

Ten years ago, I was opposed to creating special silo funds, but at this point, well… probably we need it? (a health policy expert)

I think it is necessary to clearly define budget limits for high-priced potential cures resulting in significant budget impacts. (a civic group)

Participants also mentioned the following reasons for special silo funds: *i*) to avoid making frequent exceptions in routine P&R decisions, *ii*) to diversify funding sources, *iii*) to make it easier to take actions following re-assessment (i.e., possibly easier to revise P&R conditions of those drugs funded through interim funding than those routinely funded), and *iv*) possibly to strengthen the government audit system on financing such technologies outside the NHIS.

#### Other budget-containing tools

Most stakeholders (72%) agreed to prior authorisation to prevent overprescribing high-priced potential cures. In addition, they mentioned that it could also protect healthcare providers from any financial losses by removing the possibility of reimbursement refusals. About 64% also agreed to restricting healthcare facilities where high-priced potential cures can be prescribed.

As for controlling tools towards patients, relatively fewer stakeholders agreed to this type of budget-containing tools such as increasing patients’ cost-sharing (48% agreed) and applying NHI spending caps on each patient (36% agreed).

While quite a few mentioned the need for international collaboration to better deal with the monopoly behaviour of pharmaceutical firms and the challenges brought by these new technologies, only 40% agreed to joint price negotiation, with some scepticism about the feasibility of such implementation.

Notably, throughout the FGI, many mentioned the need for strengthening (post-reimbursement) follow-up management, such as revising P&R conditions, for those drugs that did not or failed to prove their clinical and economic values at re-assessment. However, HIRA representatives complained of practical difficulties in changing the P&R conditions of those drugs already on the NHI formulary due to huge resistance possibly linked to legal disputes.

### Ways to deal with the uncertainty of high-priced potential cures

**Fig. 4:**
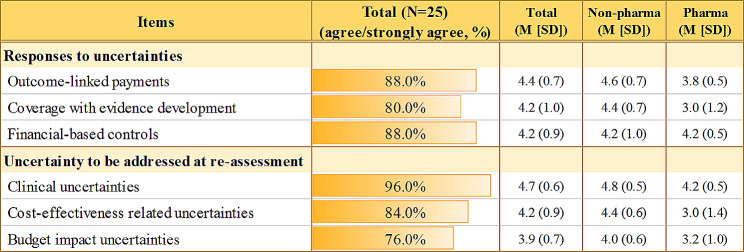
Responses to uncertainty challenages associated with high-priced potential cures. *Note* Level of agreement on the above factors was surveyed using a five-point likert scale (1 = strongly disagree, 5 = strongly agree), following focus group interviews to quantitatively summarise the stakeholders’ perspectives in a consistent manner. Values were presented as either percentages or mean (M) with standard deviation (SD), as indicated. Non-pharma includes policy & clinical experts, government payers, civic groups, and patient groups

When asked how to respond to the uncertainty challenge of high-priced potential cures, 88% agreed to outcome-linked payments, followed by financial-based controls (88%) and coverage with evidence development (80%) (Fig. 4). Pharma, particularly, preferred financial-based controls, which offer certainty in predicting their future sales and revenues. They generally presented cautious attitudes whenever the measures discussed were linked to ‘uncertainty’, in fear of the government imposing any unpredicted actions.

We also asked what type of uncertainties should be addressed at re-assessment. All but one stakeholder (96%) agreed to clinical uncertainties, followed by cost-effectiveness-related uncertainties (84%) and budget impact-related uncertainties (76%). While pharma also recognised the need for addressing clinical uncertainties at re-assessment, they were relatively more reluctant about other types of uncertainties.

Given that high-priced potential cures are likely introduced through a CEA-waiver track in South Korea, we further asked about the eligibility criteria for the track and follow-up measures. Over two-thirds (68%) agreed that the criteria should be strengthened, and more importantly, 100% (without pharma) agreed that their cost-effectiveness should be verified at re-assessment.

There is no evidence available for assessment when drugs are introduced with a CEA-waiver track. Manufacturers should submit at least something to evaluate the value of drugs… I don’t think that (the appropriateness of) paying KRW2 billion (less than US$2 million) under such circumstances has been publicly discussed in our society. (a clinical expert)

However, many stakeholders also pointed out the difficulty of implementing an effective re-assessment. To make re-assessment effective and practical, they stressed the need for an initial assessment of where uncertainties arise and an initial agreement between payers and manufacturers on how to address these uncertainties, with what outcome indicators, and how to reflect re-assessment results in P&R decision-making.

## Discussion

High-priced potential cures pose significant challenges for healthcare payers. The latest example of such technologies in South Korea, listed on the NHI formulary with a list price of nearly US$2 million, has raised real concerns over the NHI affordability and value assessment. However, there has been little public discussion about related issues. This study conducted focus group interviews and surveys with diverse stakeholders, including healthcare payers, pharmaceutical companies, health policy and clinical experts, and patient and civic groups, to help develop joint solutions to overcome these affordability and uncertainty challenges.

### Pricing arrangements

Most stakeholders still preferred confidential discounts and rebates as a default response mechanism to overcome the affordability and uncertainty challenges of high-priced potential cures, mainly because they are administratively less burdensome and can provide ‘tangible’ budget savings to the NHI. While these tools, in theory, cannot address uncertainties surrounding the values of high-priced potential cures, many stakeholders still agreed to financial controls since they can buy out some of those uncertainties while providing predictable MEA outcomes to payers and manufacturers. A minority of stakeholders even argued for relaxing eligibility criteria for such arrangements to improve the NHI’s bargaining power by allowing dual pricing (list prices and net prices protected under confidentiality agreements). In fact, differential drug pricing resulting from confidential rebates and discounts has already become a global phenomenon. Limited evidence, however, suggests that countries with larger (or influential) markets likely better exercise their bargaining power and get more discounts than those with smaller markets [7, 10]. Therefore, caution is certainly required when excessively utilising such practices or pricing based on list prices of big markets.

Outcome-based pricing arrangements also appeared to be stakeholders’ preferred option, especially as a solution to the uncertainty challenge, providing that outcomes can be clearly defined, measured, and interpreted. Pharma also presented positive attitudes towards this approach– whether they are outcome-based payments in instalments or outcome-based refunds– possibly as a strategy to penetrate their high prices into the market. Even payers agreed to outcome-based pricing arrangements despite the complexities associated with executing such arrangements. These arrangements seem to provide some relief since they can prevent, at least, a complete waste of taxpayers’ money (NHI contributions). While many stakeholders found outcome-based payments in instalments conceptually more appealing, payers had practical concerns over any pricing model involving instalment payments due to the following reasons. Firstly, the NHIS primarily reimburses healthcare providers, which are mainly private entities, instead of manufacturers. This setup inevitably requires the involvement of these providers in contracts for outcome-based instalment payments because they are the ones who purchase treatments from retailers or manufacturers and get reimbursed by the NHIS upon the use of these treatments. However, such contractual agreements pose both legal and administrative challenges within the existing NHIS framework. Therefore, adopting instalment payments is feasible only after these reimbursement challenges are addressed.

Secondly, payers expressed greater concerns over ‘cumulative liabilities’ arising from ongoing payments for previously administered (one-time or short-term) treatments than over their initial budget impacts, given the NHI’s pay-as-go system operating on an annual cycle basis (i.e., collecting contributions and spending them in the same year) (see Appendix 2 for further discussion [online supplements]). Therefore, outcomes-based refunds will likely prevail as a response mechanism to the affordability and uncertainty challenges of high-priced potential cures, just like Kymriah and Zolgensma [16, 17], the first such cases in South Korea. However, outcomes-based payments in instalments should also be ready for use, on a minimal basis, for small firms (e.g., biotech firms), inevitably having a higher risk of defaults on future refunds. A small group of healthcare providers could be designated and involved in such pricing arrangements.

While outcome-based pricing arrangements can partly address the issue of uncertainties, they still cannot justify the prices of those technologies at least in the Korean context since their cost-effectiveness has not been evaluated. In addition, not all technologies would be eligible for such resource-intensive patient-level outcome-based pricing arrangements.

### Verification of values at re-assessment

Currently, high-priced potential cures in South Korea are being introduced through a CEA waiver track for early patient access. Their prices are no longer based on HTA assessments but instead primarily on external reference pricing, subject to total expenditure caps and possibly some extra measures for very costly technologies. Their values are rarely examined as long as they meet the eligibility criteria for the track (i.e., targeting a small number of patients with cancer or rare diseases, no alternatives available, and having difficulties in evidence generation [see Appendix 1 for further details, online supplements]). However, when asked what factors should be considered in the P&R decisions of these technologies, fewer than half of stakeholders (48%) agreed to external reference pricing, mainly because public list prices in reference countries (largely big markets) are substantially higher than their net prices [21, 22], and, even if not, the net prices do not reflect Korean value judgements and country contexts.

Not surprisingly, all non-pharma stakeholders stressed the need to verify their value for money, at least, at re-assessment, especially given the substantial budget impact implications of high-priced potential cures. They also urged the need to take actions (e.g., revising P&R conditions) following re-assessment. However, payers often face huge political pressures to make innovative treatments available to patients. It seems even more challenging for payers to revise P&R conditions for those drugs already on the routine NHI formulary. As a possible solution to these challenges, reimbursement of high-priced potential cures can be made conditional or interim until their values are verified at re-assessment. For effective re-assessment and following actions, detailed terms and conditions, particularly on data collection and HTA data submission, should be pre-determined and agreed upon between payers and manufacturers before making MEA contracts. The post-2016 Cancer Drugs Fund (CDF) in England can be a good example of such a scheme in that it is made embedded in the national HTA process with clearly defined entry criteria and exit processes [1]. Under this new plan, those anticancer drugs that meet standard CE thresholds but present high uncertainties are funded through interim funding (CDF) until decisions for routine commissioning are made after completing mandatory data collection on outcome indicators identified as major sources of uncertainties [23]. Similarly, reimbursement of those high-priced potential cures could be made interim with clear entry and exit criteria in South Korea.

Notably, a CED scheme was implemented once, which was, in fact, the first case of MEA (i.e., Evoltra for acute lymphoblastic leukaemia introduced in Dec 2013) in South Korea [24]. Since then, no such arrangement has been implemented due to the complexity of data collection and related P&R agreements, partly coupled with a lack of local experience [24]. However, as the introduction of high-priced potential cures presenting high uncertainties is likely to increase in the near future, value assessment of these technologies at some point is inevitable. System environments should be adjusted to better facilitate such assessment. For instance, real-world data (RWD) will likely play a critical role in re-assessment. It would therefore be essential to improve the efficiency of RWD collection, such as bringing fragmented data sources into a centralised system. Currently, hospitals in South Korea collect detailed patient-level data, particularly for this type of potential cures, using Electronic Medical Records (EMR) systems, which are not standardised across hospitals. The Korean Ministry of Food and Drug Safety (KFDA) mandates long-term follow-up investigations for advanced biological products (e.g., 15 years for gene therapies) [25]. HIRA also collects treatment- and outcome-related data for reimbursement purposes. It may be helpful to create a centralised multi-purpose national registry, which can be linked to standardised hospital EMR systems, similar to Spanish Valtermed and Italian AIFA registries [26]. It can facilitate data collection while avoiding unnecessary duplication of efforts across regulatory bodies and hospitals.

### Special silo funds

Many stakeholders suggested the management of total health expenditures for those drugs with high uncertainties. Some (68%) agreed with creating special silo funds to set clear budget limits and avoid making frequent exceptions in routine P&R decisions. Some governments, especially in single-payer national healthcare systems, have already started to establish such funds to finance high-priced potential cures [10]. Examples include the English CDF [23] and Innovative Medicines Fund (IMF) [27], the Scottish New Medicines Fund [28], and the Australian Life Saving Drugs Program (LSDP) [29]. In England, both CDF and IMF provide interim funding for anticancer and innovative drugs until decisions for routine commissioning are made, as explained above. Meanwhile, in Australia, LSDP provides fully subsidised access to expensive essential drugs, which are found to be clinically effective but not cost-effective, for patients with ultra-rare and life-threatening diseases. While CDF and IMF act as ‘interim funding’, LSDP is a special funding programme operating on a very limited basis under equity consideration. Although our stakeholders expressed mixed opinions on creating special silo funds, this topic warrants further investigation with a broader group of stakeholders and the public audience. Whichever the case, targeting patients with high unmet needs should not be taken as a free pass for market access– those drugs that did not or failed to justify their VBPs should not be ‘routinely’ funded by the NHI.

### Limitations

This study has several limitations. Firstly, we did not clearly define high-priced potential cures but instead provided a description of these drugs’ characteristics when conducting FGIs and surveys, given that few such cases are yet available in South Korea (see Table A2-1 [online supplements]). Although we provided recent reimbursement cases with their prices, it is possible that different stakeholders had different assumptions on price thresholds when discussing the issues. Price thresholds can be particularly important for pharma, who could have different interests depending on the expected price ranges of their drugs in the pipeline.

Secondly, although this study touched on the values and issues of high-priced potential cures, we focused mainly on responses to their challenges, given that our target group is key stakeholders, not the general population. Stakeholders tended to recognise the high value of these technologies, mainly because they can offer promising opportunities for patients who would otherwise have no treatment options. It is important to understand whether these technologies deliver additional values, beyond standard VBPs, to Korean society and, if so, what attributes contribute to such values. Further research with the general population will be conducted soon based on the preliminary findings here.

Thirdly, we also presented our findings by stakeholder type (pharma vs. non-pharma) to underscore the distinct perspectives often expressed by pharma stakeholders. We incorporated pharma as one of several stakeholder groups, resulting in a sample size (*n* = 4) comparable to individual groups rather than the collective of other groups. This approach precludes our results from fully representing the entire pharmaceutical industry’s viewpoints. However, it should be noted that our pharma participants are pharma P&R staff engaged in MEA negotiations for major high-priced innovative drugs. Given the rarity yet importance of such negotiations, their insights arguably provide valuable, albeit not exhaustive, representations of industry practices. Finally, the level of understanding on study topics could have been different by the type of stakeholders, although we provided interview material in advance to help them understand related issues.

## Conclusions

This study provides an informative snapshot of payers and other stakeholders’ views on how to respond to the challenges brought by high-priced potential cures. Given their significant financial implications, several policy tools should be simultaneously applied to these technologies. Outcome-based pricing arrangements, the refunds model, in particular, should be first considered if outcomes can be measured and attributed to treatments. In addition, these drugs are likely to bypass rigorous HTA assessments at the time of launch due to limited evidence. Their values should be verified at least at re-assessment in alignment with value-based pricing principles in South Korea, especially given that those high prices can act as an anchor for the pricing of future innovations. For effective re-assessment and follow-up actions, the conditions of re-assessment should be clearly defined and agreed upon between payers and manufacturers before entering into MEA contracts. Further research is warranted to understand how much the Korean general population are willing to pay for these technologies and what aspects of such technologies contribute to additional willingness to pay, if any.

### Electronic supplementary material

Below is the link to the electronic supplementary material.

Supplementary Material 1

## Data Availability

The data that support the findings of this study are available within the article or its supplementary material.

## Abbreviations

AIFA: Italian Medicines Agency
CDF: Cancer Drugs Fund
CE: Cost-Effectiveness
CEA: Cost-Effective Analysis
CED: Coverage with Evidence Development
EMR: Electronic Medical Records
FGI: Focus Group Interview
HIRA: Health Insurance Review and Assessment Service
HRQoL: Health-Related Quality of Life
HTA: Health Technology Assessment
ICER: Incremental Cost-Effectiveness Ratio
KFDA: Korean Ministry of Food and Drug Safety
KRW: Korean Won
LSDP: Life Saving Drugs Program
LY: Life Year
MA: Marketing Authorisation
MEA: Managed Entry Agreement
NHI: National Health Insurance
NHIS: National Health Insurance Service
PBA: Performance-based Agreement
PBAC: Pharmaceutical Benefits Advisory Committee
P&R: Pricing and Reimbursement
RWD: Real-World Data
SD: Standard Deviation
US$: United States Dollar
VBP: Value-based Price
