# Supplementary material for: Values, challenges, and responses associated with high-priced potential cures: perspectives of diverse stakeholders in South Korea
Source: Cost Eff Resour Alloc. 2024 Mar 4;22:20. doi: 10.1186/s12962-024-00527-2 (PMC10913648; doi:10.1186/s12962-024-00527-2)
Supplement: Supplementary file 1 — Supplementary Material 1 [file 12962_2024_527_MOESM1_ESM.docx]

**Supplementary information**

**Manuscript title**: Values, challenges, and responses associated with high-priced potential cures: perspectives of diverse stakeholders in South Korea

# Appendix 1. Country policy backgrounds

## 1. South Korea’s healthcare system

South Korea’s healthcare system is based on the National Health Insurance (NHI) programme. Healthcare services and drugs are publicly funded through the NHI - albeit subject to substantial out-of-pocket (OOP) payments - but provided primarily by private providers through mandatory contracts with the public NHI. A sizeable private market for uninsured services also exists, which is supplied with market-based prices. Table A1-1 summarises the key characteristics of the Korean healthcare system.

### Table A1-1. Key characteristics of the Korean healthcare system

| **Type** | **Key characteristics** |
| --- | --- |
| **Health system** | - A compulsory social insurance: the National Health Insurance (NHI) system |
| **Payer** | - **Single-payer system** governed by the National Health Insurance Service (NHIS) |
| **Population coverage [1]** | - **100%** (97% covered by NHI + about 3% covered by Medical Aid Programme [MAP – for those disadvantaged]) |
| **NHI financing [2]** | - KRW 85.5 trillion: NHI contributions etc. (86%) + government subsidies |
| **Contributions [3]** | - **The employee insured**: monthly contribution = monthly wage **x** contribution rate (7.09% in 2023) (equally shared between the employee insured and their employers) - **The self-employed insured** (the rest group): monthly contribution = contribution score **x** value per score (contribution scores based on income, property, and car ownership) - (**Medical aid beneficiaries**: no contributions) |
| **Service coverage [2]** | - **64.5%** as of 2021 (= NHI expenditures/[NHI expenditures + patients’ NHI cost-sharing [4]^a^ + patients’ out-of-pocket payments for those services not covered by NHI]) - (^a^**Annual limits** applied to patients’ NHI cost-sharing.) |
| **Type of service providers [5]** | - **Mainly private** (94.7% of hospitals; 90.4% of hospital beds) → contracts between the public NHI and (mostly) private providers for the delivery of services (contract opt-out not possible) |
| **Provider payment** | - Mainly **fee-for-service** based |

There are three key government bodies operating the health system: the Ministry of Health and Welfare (MoHW) and its two fundamental institutions, the National Health Insurance Service (NHIS) and the Health Insurance Review & Assessment Service (HIRA).

- The MoHW oversees the NHI system and works on national health policies.
- NHIS, serving as the single public insurer, gathers NHI contributions and compensates healthcare providers for the portion of health expenditures not directly covered by the patients (i.e., cost sharing), based on a predetermined fee schedule.
- HIRA conducts claims reviews prior to the provider payments and quality assessment of healthcare services. It also assesses the appropriateness of reimbursing healthcare services and drugs.

Figure A1-1 depicts how healthcare services are provided under the NHI system with the role of each actor.

### Figure A1-1. South Korea’s NHI system


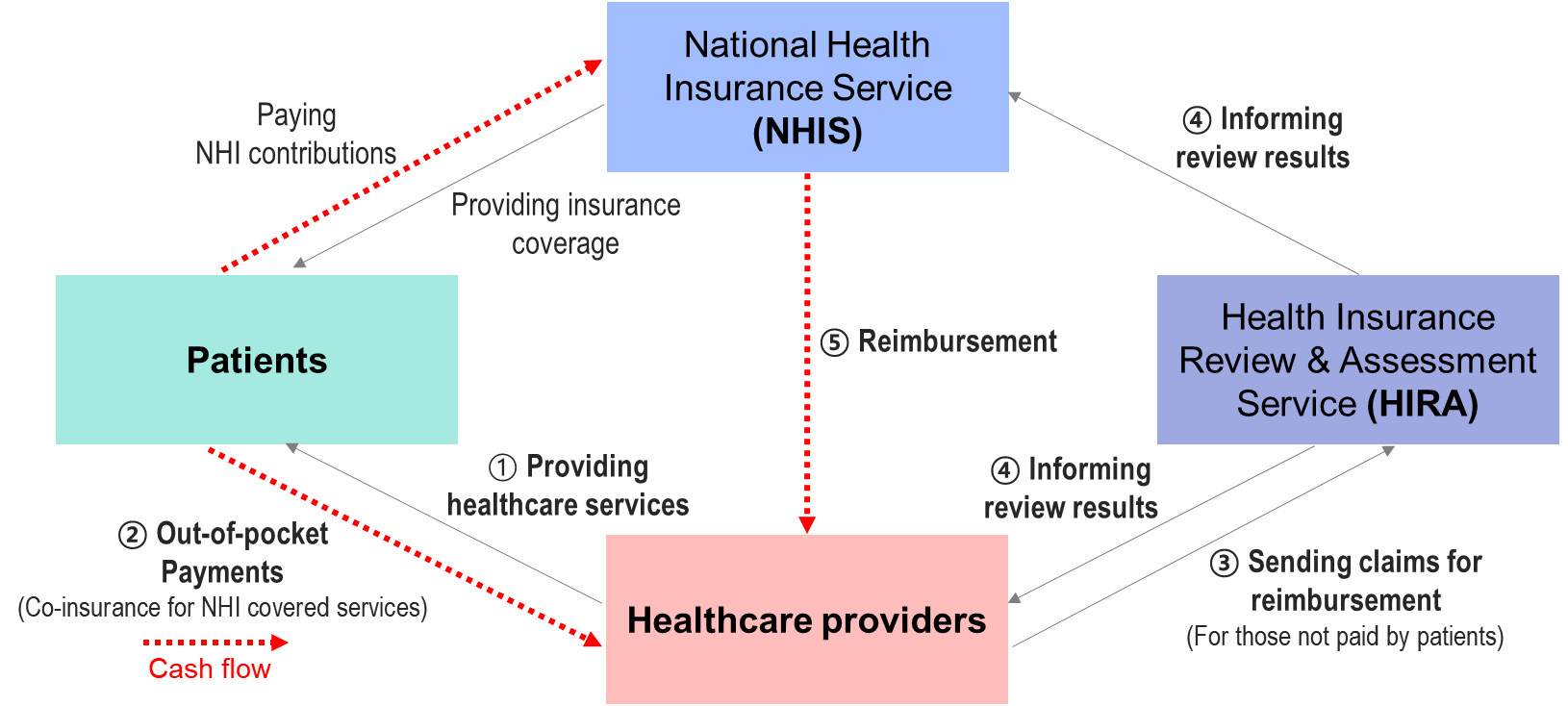


## 2. Drug pricing and reimbursement system in South Korea

### 1) Introduction of a positive listing system for new drugs

South Korea implemented a positive listing system policy on 31 December 2006 to enhance the efficiency of healthcare resource allocation. This system mandates that manufacturers submit cost-effective analysis (CEA) data to secure a premium listing for their new drugs on the NHI formulary^[[1]](#footnote-1)^.

HIRA reviews Health Technology Assessment (HTA) submission dossiers and makes a reimbursement decision largely based on clinical effectiveness and cost-effectiveness. Those drugs presenting an incremental cost-effectiveness ratio (ICER) of less than approximately KRW25 million (about US$20,800) [6] (KRW50 million for anti-cancer drugs) were implicitly considered cost-effective. However, HIRA has recently announced that they do not have any explicit/implicit ICER thresholds and will consider past ICER outcomes when making a reimbursement decision. The median ICER values of past HTA submissions during 2007-2021 are KRW15.97m for common drugs (neither anti-cancer nor orphan drugs), KRW45.16m for anti-cancer drugs, KRW32.32m for orphan drugs [7].

As shown in Figure A1-2, once HIRA makes a positive recommendation for reimbursement, NHIS negotiates a list price and other budget impact implications with manufacturers.

### Figure A1-2. The NHI listing system for new drugs


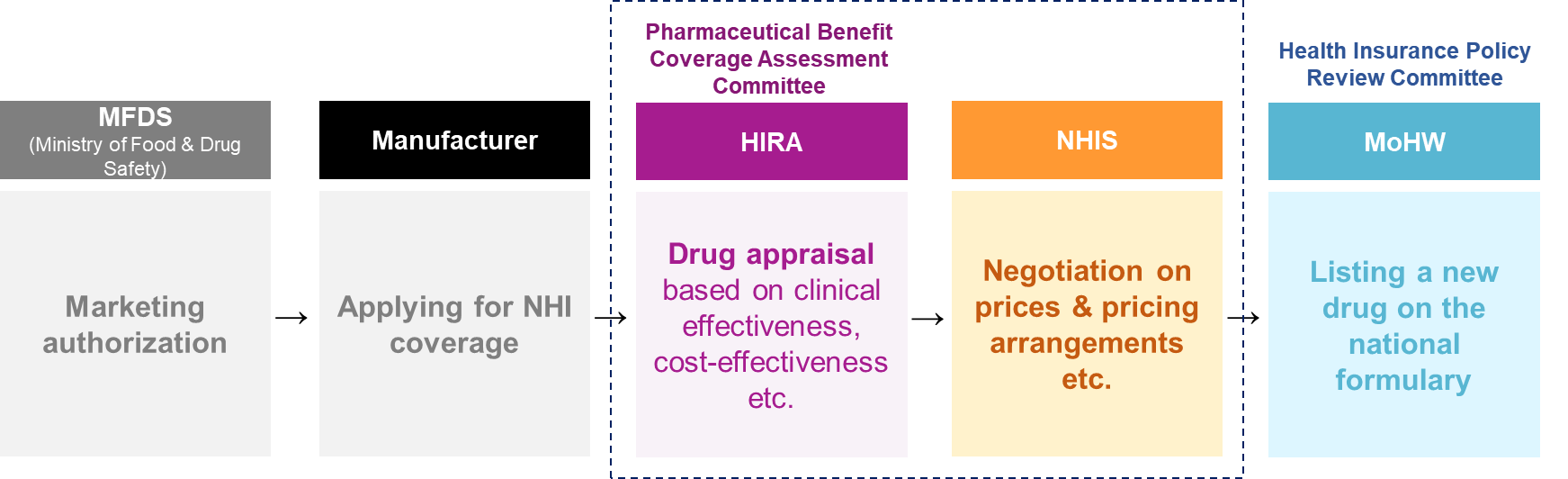


Drugs are provided under the NHI system, similar to other healthcare services, as depicted in Figure A1-3.

### Figure A1-3. South Korea’s drug delivery and reimbursement system under NHI


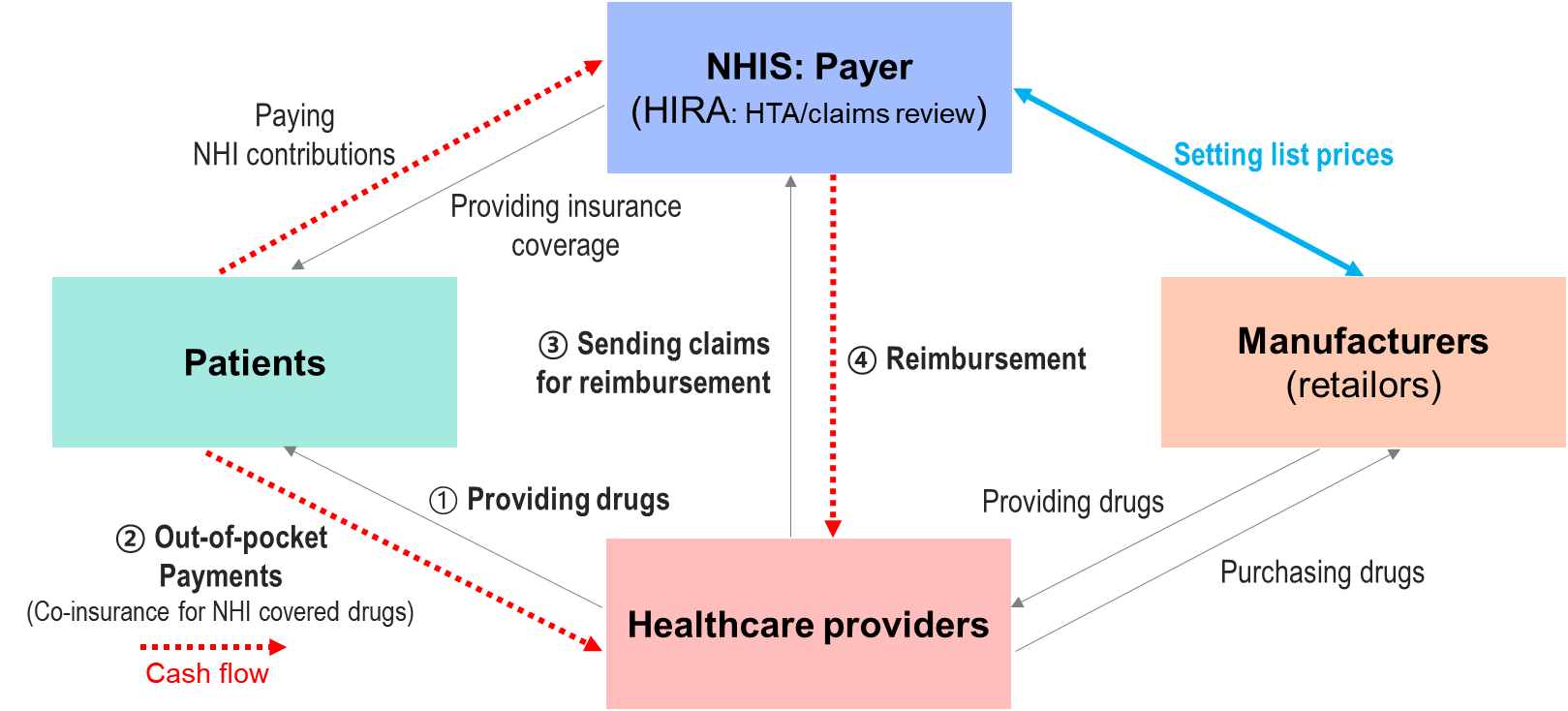


### 2) New pathways for listing drugs

To enhance early patient access to innovative drugs for conditions with significant unmet medical needs, the government introduced new pathways, including the risk-sharing arrangement (RSA) and CEA-waiver track.

**(1) Risk-Sharing Arrangements (RSA) track**

Launched in December 2013, the RSA track is specifically designed for drugs addressing all types of cancers or rare, life-threatening diseases lacking therapeutic options. RSAs encompass various subtypes, including refunds, expenditure caps, utilisation caps per patient, and, most recently, outcome-based pricing arrangements. The refund type, notable for implicitly facilitating dual pricing (whereby list prices and net prices can differ based on refunds), emerges as the most prevalent model. Despite the adaptability RSAs offer, drugs pursuing these arrangements are still mandated to submit HTA dossiers. This requirement ensures that the drugs not only meet the immediate needs of patients but also adhere to standards of cost-effectiveness and clinical effectiveness, qualifying them for price premiums.

**(2) CEA waiver track**

Given the recognition that drugs requiring early patient access often have limited clinical data available at the time of launch, the government introduced the CEA waiver policy in February 2015. Currently, the following anticancer or orphan drugs targeting a small number of patients are eligible for this track[8]:

1) **a.** no alternative treatments available, **b.** no therapeutically equivalent treatments available and targeting life-threatening conditions, OR **c.** no therapeutically equivalent treatments available for paediatric conditions and providing clinically meaningful quality of life improvement (added in December 2022); AND

2) **a.** marketing authorisation obtained with single-arm trial data, **b.** marketing authorisation obtained with phase 2 clinical trial data without the condition of conducting phase 3 trial data, OR **c.** evidence generation difficult for other reasons that the committee agree to; AND

3) listed in three or more of the A8 reference countries (A8 countries: Canada [added in December 2022], France, Germany, Italy, Japan, Switzerland, the UK, and the US).

In 2020, the eligibility criteria were further expanded to include those essential tuberculosis drugs, antibacterial drugs, and emergency antidotes publicly available in three or more of the reference countries and introduced without CEA data in these countries or judged to be challenging to conduct CEA even if such data are available elsewhere.

The price for drugs under the CEA waiver track is set based on the lowest price of the A8 adjusted prices (ex-factory prices: 73% [UK] ~ 93% [Italy]). Since September 2016, these drugs are also subject to expenditure caps under RSA, with the possibility of applying additional RSA tools through negotiations between NHIS and manufacturers. This pricing strategy extends to new drugs that are therapeutically equivalent to earlier introductions through the CEA waiver track.

# Appendix 2. Additional information on focus group interviews and surveys

## 1. Characteristics of ‘high-priced potential cure’

Table A2-1 describes the characteristics of high-priced potential cures recently introduced in South Korea. Rather than clearly defining these drugs, we provided this description to study participants when conducting focus group interviews and surveys.

### Table A2-1. Characteristics of high-priced potential cures

| **Factors** | **Characteristics** |
| --- | --- |
| Clinical benefits | - Potentially cure disease, although the definition of cure varies. - However, clinical effectiveness is uncertain due to immature clinical evidence at the time of launch. |
| Budget impacts | - Ultra-expensive, often high upfront costs (i.e., high initial budget impact) required for one-time or short-term treatment (e.g., gene therapies), threatening health care affordability in the short- and medium-term and sustainability in the long-term. |
| Value for money | - Value for money is uncertain due to a lack of long-term clinical and economic evidence. |
| Other | - Providing treatment alternatives to patients with life-threatening conditions who would otherwise be left without relevant treatment options. - No further treatment is required if it is a one-time treatment. - R&D costs are likely very high (mass production is challenging). - Often targeting rare conditions (normally 10~200 patients per year, possibly up to 1,000 patients) - Often targeting paediatric conditions |

## 2. Outcome-linked payments: payment upon success and refunds upon failure

While outcomes-based payments in instalments are conceptually appealing to many stakeholders, compared to outcomes-based refunds, practical concerns from payers under the Korean NHI system have surfaced. In this system, the NHIS, acting as the single payer, reimburses healthcare providers, mainly private entities, who purchase and prescribe treatments. The feasibility of outcomes-based payments in instalments hinges on resolving reimbursement processes since the NHIS reimburses healthcare providers and not directly the manufacturers.

In addition, payers express significant worries regarding ‘cumulative liabilities’—the financial burden of ongoing payments for previously administered treatments—given the NHI’s pay-as-you-go model, which operates on an annual cycle of collecting and spending contributions. This concern is contrasted with the immediate budget impact of (one-time or short-term) new technologies.

From a patient perspective, outcomes-based refunds might be preferable, as mandatory OOP payments are made only once upfront, with annual limits capping the cost regardless of the treatment’s price. Conversely, outcomes-based payments in instalments could result in multiple OOP payments over time, potentially increasing the total financial burden on patients despite the application of annual limits to each payment.

Nonetheless, there is a recognition that outcomes-based payments in instalments should be available, at least minimally, for small firms, such as biotech companies, which face a higher risk of defaulting on future refunds. This approach could involve a select group of healthcare providers in such pricing arrangements, addressing the risk of defaults while still promoting innovation.

Table A2-2 compares both types of outcome-based payments, especially regarding financial implications, from different actors’ perspectives.

### Table A2-2. Financial considerations for outcome-linked payments under the NHI system

| **Factors** | **Outcomes-based**  **payments in instalments** | **Outcomes-based**  **refunds** |
| --- | --- | --- |
| **Level of initial budget impacts**  (For one-time or short-term treatment) | - **Low**: it is possible to spread out the initial budget impacts across several years, but this approach can raise ‘cumulative liabilities’ arising previously administered treatments, limiting the payer’s flexible responses to future financial conditions, especially given the NHI’s pay-as-go system operating on an annual basis. | - **High** - (**Note:** The initial budget impact can be less of a concern under the single-payer system.) |
| **Budget/fiscal considerations** | - **More complex** - (Instalment type of payments requires changes to accounting rules to allow for payments outside in-year budget cycles.) | - **Simpler** - (Refunds taken as ‘other revenues’) |
| **Debtors**  (Treatment liabilities) | - **Payer** (government) - **Patients** (cost-sharing) | - **Manufacturers** |
| **Risk of debtors** | - **Single payer: low** - **Patient (for one-time or short-term treatment): possibly high** if patients have to make their out-of-pocket (OOP) payments (cost-sharing) over time for their past treatment | - **Manufacturer:** - **Low** if it is a large-size global firm, but **possibly high** if it is a small-size firm (e.g., biotech company) due to higher risk of bankruptcy or change of ownership over time |
| **Patients’ perspective**  (Annual limits applied to patients’ cost-sharing) | - **Paying more** - (Patients’ OOP payments could increase with the number of instalments over time because patients’ cost-sharing for each instalment could exceed annual limits on OOP spending.) | - **Paying less** - (Patients face only one-time cost-sharing when taking treatment, subject to an annual limit. Patients will likely pay up until the limit, regardless of treatment prices.) |
| **Healthcare providers’ perspective** | - **More complex** - (Need to consider how to reimburse healthcare providers under this pricing arrangement since they are the ones who purchase and prescribe treatments. Otherwise, it will cause a temporal misalignment between providers’ payment for purchasing treatment and receiving reimbursement for prescribing treatment.) | - **Less complex** - (No need to consider healthcare providers under this pricing arrangement since manufacturers will refunds directly to the payer.) |
| **Manufacturers’ perspective** | - R&D costs cannot be immediately recouped (-). | - The present value of receiving the lumpsum payment now is greater than that of receiving instalments over time (+). - VAT, applied to initial list prices, is not refunded even if refunds upon failure are made (-). |

# References

[1] Korean Statistical Information Service. Yearly NHI population coverage status 2023. Available at: <https://kosis.kr/statHtml/statHtml.do?orgId=350&tblId=TX_35001_A001> [accessed 2023.20 February].

[2] Statistics Korea. NHI financing and coverage rates. 2023. Available at: <https://www.index.go.kr/unity/potal/main/EachDtlPageDetail.do?idx_cd=2763> [accessed 2023.20 February].

[3] National Health Insurance Service. Contribution rates. 2023. Available at: <https://www.nhis.or.kr/english/wbheaa02500m01.do> [accessed 2023.20 February].

[4] National Health Insurance Service. Information on level of mandary patients' NHI cost-sharing. 2023. Available at: <https://www.hira.or.kr/dummy.do?pgmid=HIRAA030056020100> [accessed 2023.20 February].

[5] Korean Statistical Information Service. Percentages of public healthcare institutions by province 2023. [accessed

[6] Lee IH, Bloor K, Bae EY. A Comparative Analysis of Anticancer Drug Appraisals Including Managed Entry Agreements in South Korea and England. Appl Health Econ Health Policy 2023;21:347-59.

[7] Assessment HIRa. The summary of CE results (ICER) for drugs with HTA submission dossiers (2007-2021). 2022. Available at: <https://www.hira.or.kr/bbsDummy.do?pgmid=HIRAA020002000100&brdScnBltNo=4&brdBltNo=10021&pageIndex=1#none> [accessed 2023.20 February].

[8] Health Insurance Review and Assessment. Regulations on evaluation criteria and procedures for deciding reimbursement eligibility criteria (약제의 요양급여대상여부 등의 평가기준 및 절차 등에 관한규정 in Korean). Wonju: HIRA; 2022. Available at: <https://www.hira.or.kr/bbsDummy.do?pgmid=HIRAA040055000000&WT.gnb=%EC%8B%AC%EC%82%AC%ED%8F%89%EA%B0%80%EC%9B%90%EB%82%B4%EB%B6%80%EA%B7%9C%EC%A0%95> [accessed 2023.02.20].

1. For a new drug that is judged to be more clinically effective than its alternative (i.e., normally the one with the highest market share) but has not submitted CEA data, its price is set based on 110% of the highest price of its alternatives (10% additional). For a new drug that is found to be clinically effective, similar to its alternative (non-inferior), its price is set based on the weighted average price (WAP) of its alternatives. [↑](#footnote-ref-1)
